# Fiber Synergy of Polyvinyl Alcohol and Steel Fibers on the Bond Behavior of a Hybrid Fiber-Reinforced Cementitious Composite

**DOI:** 10.3390/ma17030629

**Published:** 2024-01-27

**Authors:** Wenlin Liu, Jianping Han

**Affiliations:** 1 School of Urban Construction and Transportation, Hefei University, Hefei 230601, China; 2 Key Laboratory of Disaster Prevention and Mitigation in Civil Engineering of Gansu Province, Lanzhou University of Technology, Lanzhou 730050, China; jphan@lut.edu.cn; 3 Institute of Earthquake Protection and Disaster Mitigation, Lanzhou University of Technology, Lanzhou 730050, China

**Keywords:** fiber synergy, cracking process, hybrid fiber, hybridization, bond behavior

## Abstract

Based on multi-scale characteristics inherent in the cracking process of cementitious composites, fibers with different geometric dimensions are simultaneously used to restrain the formation and development of cracks at different scales. Accordingly, hybrid fiber-reinforced cementitious composites (HyFRCCs) exhibit excellent bond behavior and deformation capacity in terms of tension and compression, accompanied by higher damage tolerance. Using these benefits of the mechanical properties of HyFRCCs, the structural performance of HyFRCC structures under complex loading conditions can be improved. To objectively evaluate the contributions of all fibers to the mechanical properties of HyFRCCs, steel macro-fibers, and polyvinyl alcohol (PVA) micro-fibers were used to design several reinforced cementitious composites. Four of the specimens were mono-fibrous cementitious composites, three specimens were cementitious composites reinforced with hybrid fibers, and one was a non-fibrous cementitious composite. The synergy effect of the steel and PVA fibers was analyzed using various fiber combinations. The results indicated a significant enhancement of the bonding properties of HyFRCCs through the incorporation of PVA and steel fibers. Specifically, the peak bond strength, peak slip displacement, and residual bond strength exhibited increments ranging from 31.0% to 41.7%, 60.6% to 118.4%, and 34.6% to 391.3%, respectively, in comparison to the reference test block. Notably, the combined presence of the PVA and steel fibers consistently demonstrated a positive confounding effect on the residual bond strength. However, negative confounding effects were observed in terms of the peak bond strength and peak slip displacement, particularly with 1.0% steel fiber content and 0.5% PVA fiber content.

## 1. Introduction

The brittleness inherent in traditional concrete severely limits the applicability and safety of concrete structures, particularly under highly complex loading conditions. A solution to the brittleness and poor anti-cracking performance of concrete lies in the incorporation of short, randomly distributed fibers [1]. Liu et al. achieved a remarkable increase of approximately 86% in the splitting tensile strength of concrete by adding short steel fibers [2]. Notably, polyvinyl alcohol fiber (PVA) significantly influences the performance of cementitious composites [3]. Rahmati et al. highlighted that the introduction of 2% PVA fibers resulted in substantial improvements in the maximum compressive strength of concrete, reaching values of 17% and 56% [4]. The incorporation of fibers yields substantial enhancements in both the tensile and compressive characteristics of concrete. Moreover, the seismic performance of structures reinforced with fibers experiences a significant improvement.

Despite these notable advancements, the development of cracks in concrete remains a complex, multi-scale, and continuous process [5]. Many types of fiber-reinforced concrete typically incorporate only a single type of fiber, limiting their effectiveness in restraining crack development to within a specific range of crack opening and deflection [6,7,8]. Engineered cementitious composites (ECCs) find widespread use in civil engineering projects due to their responsibility for multiple cracking behaviors and strain-hardening characteristics [9]. However, the compressive toughness of ECCs decreases dramatically as their strength increases, which is attributed to the smaller dimensions and higher pull-out rate of PVA fibers. Achieving comprehensive control over crack formation proves challenging when employing a single type of fiber [10].

Harnessing synergies among fibers of varying scales, hybrid fiber-reinforced cementitious composites (HyFRCCs) demonstrate exceptional ductility and the capability for comprehensive crack control [11]. Teng et al. emphasized substantial improvements in the mechanical qualities of concrete through the combination of steel and PVA fibers. In fiber-reinforced concrete, the use of steel fibers effectively enhances flexural resistance and toughness after cracking. However, flexural strength is decreased when a combination of PVA and double hooked-end steel fibers is used [12]. Singh et al. observed that despite the limited independent impact of polypropylene fibers on the flexural strength of concrete, their role in enhancing the bending strength became remarkably significant within a steel–polypropylene hybrid fiber system. Furthermore, the study revealed that at elevated fiber content levels, the uniform distribution of both fibers in the concrete led to a reduction in compressive strength due to the mixing of fibers [13]. During the axial compression testing of steel–basalt hybrid fiber-reinforced concrete, Khan et al. observed that the introduction of basalt fibers resulted in enhanced compressive strength and toughness in steel-fiber-reinforced concrete. However, as the basalt fiber content was progressively increased, the compressive strength and toughness of the hybrid fiber-reinforced concrete exhibited a subsequent decline [14]. Deng et al. conducted an in-depth exploration into the uniaxial cyclic tension performance of concrete reinforced with steel and polypropylene fibers. Their findings revealed the substantial impact of steel fibers on enhancing cyclic tensile properties, improving post-peak residual strength, and enhancing toughness. Simultaneously, polypropylene fibers contribute to enhancing the deformation and energy dissipation capacity in the matrix [15]. The impact resistance of basalt–barchip hybrid fiber-reinforced concrete demonstrates notable improvements in both the initial strength and energy dissipation capacity of the material [16]. In a related study, Liao et al. [17] successfully developed a cost-effective HyFRCC by incorporating polypropylene and basalt fibers, thereby replacing a portion of the PVA fibers. Their investigation encompassed an analysis of the compressive, tensile, and impact resistance properties. The results revealed a substantial increase in the post-peak compressive toughness, tensile strength, and initial cracking impact strength of the HyFRCC with higher proportions of polypropylene and basalt fibers. Conversely, the performance of the ECC exhibited a slight decline due to the elevated PVA fiber content.

Despite previous research indicating significant advancements in the mechanical properties of hybrid fiber-reinforced concrete using various fiber combinations [18,19], it is essential to emphasize that the synergistic impacts of different fibers may not always result in a cumulative improvement (1 + 1 > 2). In an investigation conducted by Pakravan et al. [20] on a PP/PVA–PVA HyFRCC, the combination of fibers had an adverse effect on both the initial crack bending toughness and peak bending toughness of the composite material. This suggests that the mere inclusion of diverse fibers may not consistently enhance the concrete’s performance. Similar negative reinforcement effects between fibers were noted by Banthia et al. [21] and Chasioti et al. [22] in flexural tests on steel–cellulose hybrid fiber concrete and hybrid steel fiber concrete, respectively.

The properties of HyFRCCs have undergone extensive investigation, yet our comprehension of the synergistic properties among the fibers within their matrixes remains notably limited. Unfortunately, there is a lack of available data to assess the impact of fiber synergy on the bond behavior of HyFRCCs under direct pull-out loading. Accordingly, a combination of macro-steel fibers and micro-PVA fibers was incorporated into the design of multiple reinforced cementitious composites in this investigation. The bond behavior of HyFRCCs with various fiber combinations was thoroughly investigated using direct pull-out tests. Subsequently, a comprehensive analysis was undertaken to evaluate the impact of fiber synergy on the bond strength and bond toughness of HyFRCCs. Here, our objective was to elucidate the synergistic mechanism between various fibers in HyFRCCs. This was achieved through a quantitative analysis of the combined effect of PVA fibers and steel fibers in a PVA–steel HyFRCC. The findings of this analysis aim to establish a foundation for the optimal design and application of HyFRCCs.

## 2. Experimental Program

### 2.1. Material Composition and Mix Ratio

In accordance with the design and construction guidelines for ECCs, as well as the findings of earlier research [23,24], the formulation of the HyFRCC mix ratio was devised. All the investigated mixtures shared identical components, with variations limited to the selection and arrangement of fibers. The mixture consisted of ordinary Portland cement (P.O. 42.5), standard sand, and class II fly ash. Table 1 provides a detailed quantitative breakdown of each component in the mixture, excluding fibers.

**Figure 1:**
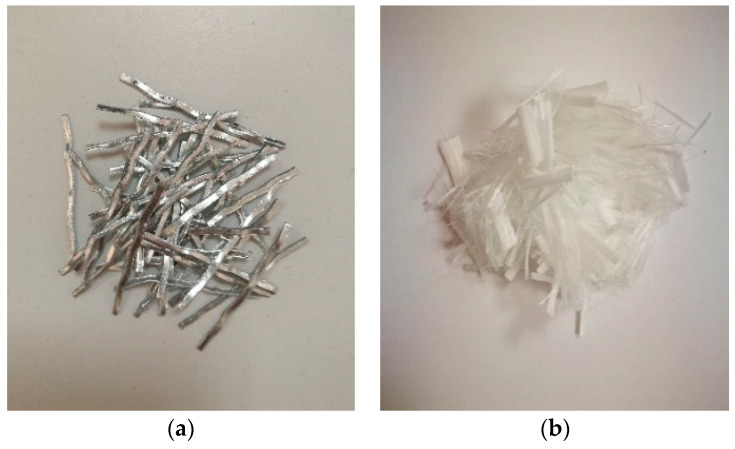
The fibers investigated for bond–slip behavior: (**a**) steel fibers, (**b**) PVA fibers.

The steel fibers and PVA fibers investigated in this paper are shown in Figure 1. The properties of the steel fibers and PVA fibers are shown in Table 2 and Table 3. Additionally, the PVA fibers employed in this research were manufactured by Kuraray.

### 2.2. Specimen Design

Eight groups of specimens were designed and investigated, as outlined in Table 4. These comprised one group of plain concrete labeled as S0PA0. Among the other groups, four were mono-fibrous cementitious composites, denoted as S0PA10, S10PA0, S0PA15, and S15PA0. Additionally, three groups were HyFRCCs, named S05PA05, S05PA10, and S10PA05. The sole difference among these groups lies in the combination and content of fibers.

**Figure 2:**
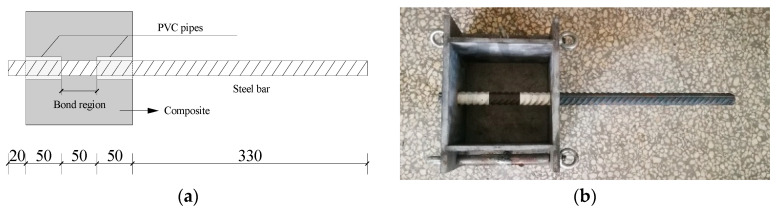

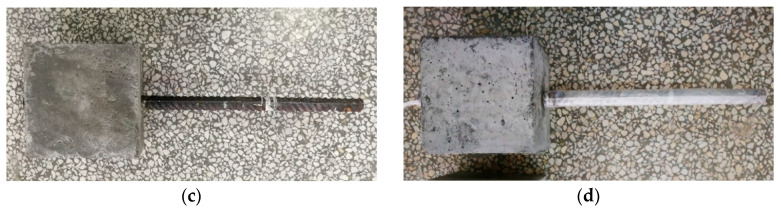
Specimen for direct pull-out test (Unit: mm): (**a**) specimen size, (**b**) casting mold, (**c**) direct pull-out specimens, and (**d**) rust prevention treatment of steel bars.

The research outcomes reported by Li et al. reveal that, under normal temperature conditions, a bond length ranging from 2 to 3 times the diameter of the steel bar results in a typical shear failure mode during the direct pull-out test [25]. For comparative analysis, a piece of steel rebar with a diameter of 20 mm was partially embedded in a HyFRCC cube measuring 150 × 150 × 150 mm, with the embedded length set at 2.5 times the rebar diameter. The bonding region was situated in the specimen’s middle. To prevent stress concentration-induced local failure at the ends, plastic pipes covered the steel rebar, preventing bonding between reinforcement and composite. The details of the direct pull-out specimens are displayed in Figure 2a–c.

Each group involved the casting of three test specimens in steel molds, which were compacted using a vibration table. After casting for 24 h, each specimen was de-molded and cured under standard circumstances for another 27 days in a room maintained at 20 ± 2 °C and 95% relative humidity. To prevent steel corrosion, plastic wrap was tightly applied to the exposed steel rebar, as depicted in Figure 2d.

### 2.3. Loading Device and Testing Procedure

**Figure 3:**
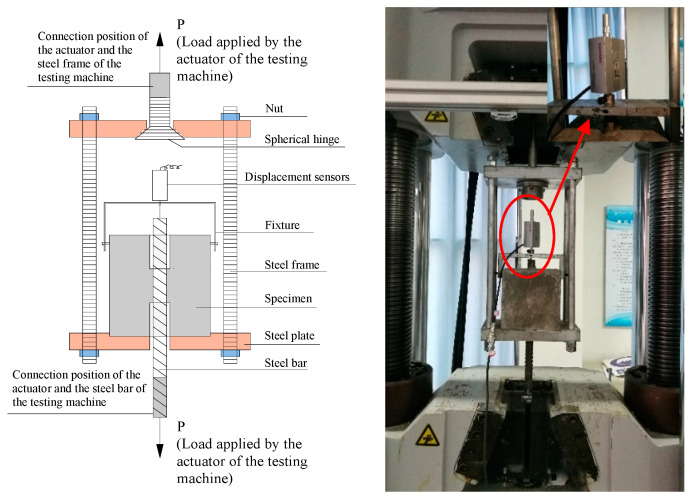
Direct pull-out test setup.

The pull-out test setup is shown in Figure 3. Each specimen was firmly affixed to the test machine through a custom-made, mild steel frame. Employing an electro-hydraulic servo universal testing apparatus boasting a load capacity of 2000 kN, the pull-out tests were executed at a loading displacement rate of 1 mm/min. The upper and lower actuators of the testing machine anchored one end of the steel bar and the upper end of the steel frame, respectively, to apply tension to the steel bar. The steel bar slip was measured with a grating displacement sensor positioned at the free end of the specimens, and the load cell embedded in the test machine recorded the pull-out force.

## 3. Results and Discussion

**Figure 4:**
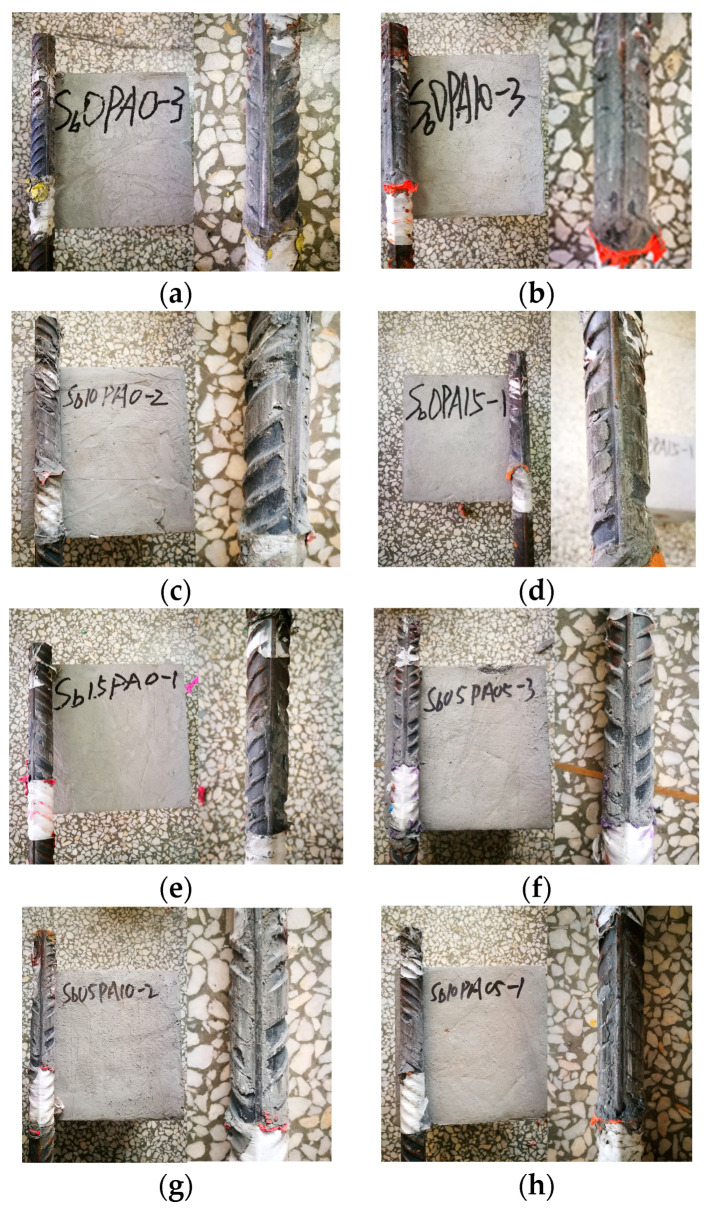
Bond–slip failure mode of specimens: (**a**) S0PA0, (**b**) S0PA10, (**c**) S10PA0, (**d**) S0PA15, (**e**) S15PA0, (**f**) S05PA05, (**g**) S05PA10, and (**h**) S10PA05.

The bond–slip failure modes between reinforcement and concrete can be classified into three types: shear failure, shear splitting, and splitting failure. As illustrated in Figure 4, no cracks were observed at the loading end or the surface of the test block, and all test specimens remained intact after failure during the direct pull-out test. Due to the ample constraints offered by the protective layer thickness of the longitudinal steel bar and the bridging effect of fibers on cracks at the interface between reinforcement and matrix, all specimens exhibited the characteristic shear failure mode.

### 3.1. Bond Force–Slip Curves

**Figure 5:**
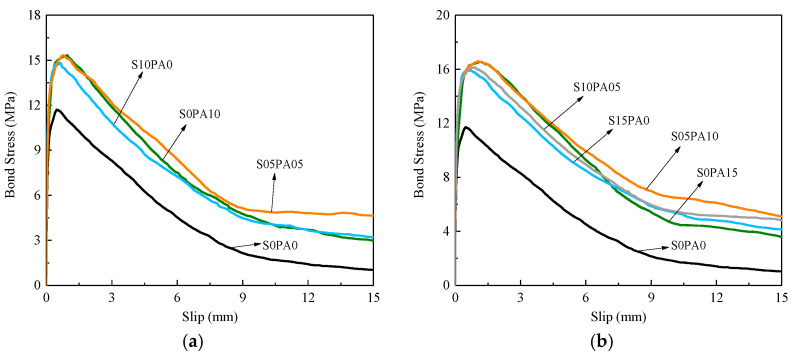
Bond stress–slip curves of specimens: (**a**) 1.0% total fiber volume fraction, (**b**) 1.5% total fiber volume fraction.

Utilizing the test results, the bond force–slip curves for each specimen were deduced and are illustrated in Figure 5. Notably, the bond force–slip curves of fiber-reinforced cementitious composite specimens exhibited a more comprehensive profile compared to non-fibrous specimens. Moreover, owing to the effective control of fiber on the cracks at the interface of the rebar and matrix, both the ultimate and residual bond strength demonstrated a significant increase in comparison to the reference specimens. In addition, in the PVA–steel hybrid fiber system, two fibers of different scales were able to effectively control cracks of different widths, compensating for the limitations of single-doped fibers in crack control. The bond stress–slip curves of the HyFRCC specimens were fuller and smoother than that of single-doped fibers. By comparing the bond stress–slip curves of the single-doped PVA fiber and single-doped steel fiber specimens, it can be seen that, with the same fiber content, the small-scale PVA fiber has better control over micro-cracks than the large-scale steel fiber, and the peak bond stress and corresponding slip of single-doped PVA fiber specimens are significantly greater than that of single-doped steel fiber specimens. However, the effective control of cracks with larger widths exhibited by the steel fiber led to the post-peak bond stress–slip curve of the single-doped steel fiber being gentler than that of the single-doped PVA fiber.

### 3.2. Bond Strength

Assuming that the bond force is uniformly distributed along the bond length, the bond stress during the pull-out testing can be calculated via Equation (1):

(1) $$\tau =\frac{P}{\pi dl}$$

where *τ* is the bond stress, *P* is the pulling force, and *d* and *l* represent the diameter of the steel bar and bond length, respectively. Both the ultimate and residual bond strength ($\tau_{u}$ and $\tau_{r}$) were calculated using Equation (1), and the corresponding results are presented in Table 5.

Table 5 presents a substantial improvement in the ultimate and residual bond strength of the cementitious composite with the introduction of fibers. The ultimate bond strength, peak slip drift, and residual bond strength of the fiber-reinforced specimens exhibited increments ranging from 26.56% to 41.67%, 30.40% to 144.03%, and 180.77% to 391.35%, respectively, in comparison to the benchmark specimens. These enhancements stem from the bridging effect of fibers within the matrix. Nevertheless, the impact varied with fibers of different geometric sizes. While maintaining the same fiber content, the escalation in ultimate bond strength and peak slip drift was more pronounced for PVA fiber than for steel fiber. Conversely, the increase in residual bond strength for PVA fiber was weaker compared to that of steel fiber. This discrepancy primarily arises from the nature of micro-cracks in the matrix prior to the peak load, where PVA fiber demonstrated superior control over smaller-scale cracks than steel fiber. Subsequent to the peak load, the development of micro-cracks progresses into macro-cracks in the matrix. During this phase, PVA fibers within the cracks underwent pull-out or breakage from the matrix, proving challenging in inhibiting macro-crack development. In contrast, steel fibers effectively impeded macro-crack progression. Surprisingly, the bond strength of HyFRCCs did not consistently surpass that of the mono-fibrous cementitious composites under constant total fiber content. For instance, when the total fiber content was 1.0%, the residual bond strength of the HyFRCC specimen S05PA05 surpassed that of a single PVA-fiber-reinforced specimen by 55.18%, while the peak slip displacement was 26.34% lower than that of the single PVA fiber-reinforced specimen. This suggests the existence of both “positive” and “negative” synergy between fibers of different geometric sizes in the hybrid fiber-reinforced cementitious composites.

### 3.3. The Effects of Parameters on Bond Behaviors

#### 3.3.1. Ultimate Bond Strength

**Figure 6:**
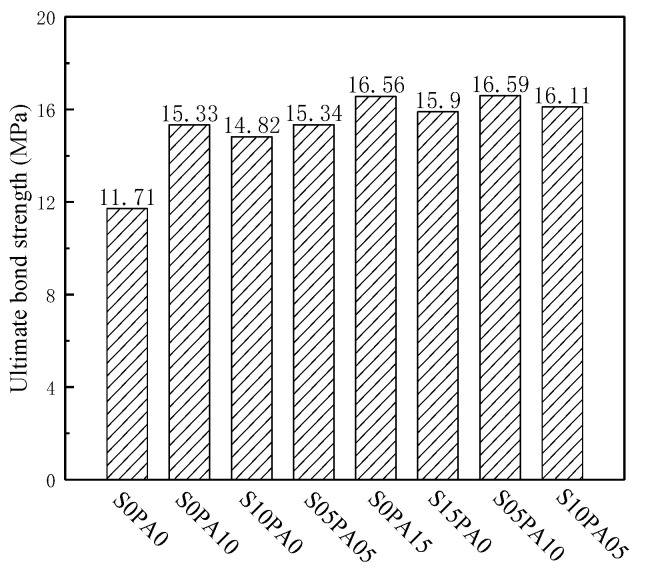
Ultimate bond strength of specimens.

The introduction of fibers resulted in a significant increase in the peak bond strength of the test block. Micro-cracks predominated at the rear-matrix interface when the load did not reach its peak. As large-scale steel fibers had a limited effect in hindering the formation and progression of micro-cracks, the enhancement of peak bond strength for steel fibers was not prominently evident. Figure 6 illustrates that the improvement in peak bond strength for PVA fibers surpassed that of steel fibers. Maintaining a constant fiber content (*V_f_* = 1.5%), the ultimate bond strength of the S10PA05 specimen decreased by 2.7% compared to the S0PA15 specimen. The decline in the ultimate bond strength of the S10PA05 specimen could be attributed to steel fiber’s insufficient control capacity of micro-cracks. In contrast, the peak bond strength of the S05PA10 specimen increased by 0.2% and 4.3% compared to the S0PA15 and S15PA0 specimens, respectively, suggesting a favorable synergistic effect between fibers on the peak bond strength when *V*_PVA_ = 0.5% and *V*_S_ = 1.0%. This was attributed to the ability of large-scale steel fibers to pass through multiple micro-cracks simultaneously, coordinating the stress at defects in the matrix across regions and enhancing the PVA fibers’ control of micro-cracks.

#### 3.3.2. Residual Bond Strength

**Figure 7:**
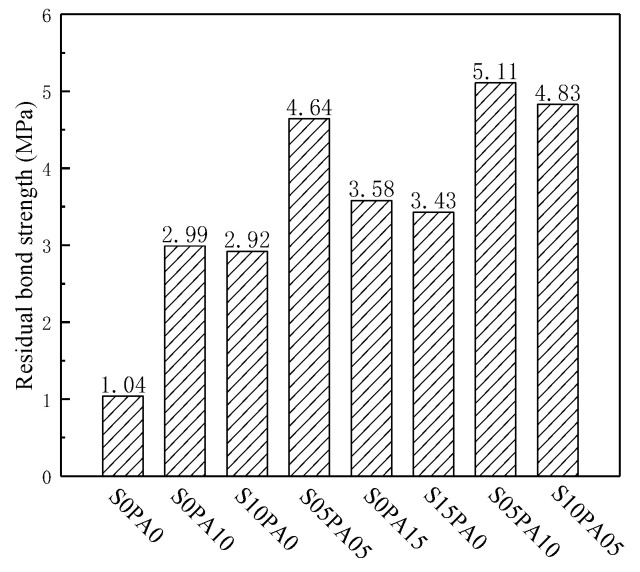
Residual bond strength of specimens.

With an increase in slip displacement, damage at the steel–matrix interface accumulates, leading to the propagation of micro-cracks that coalesce into large-scale macroscopic cracks. Under these conditions, steel fibers exhibit effective inhibition of large-scale crack development. As illustrated in Figure 7, the improvement in residual bond strength for steel fibers significantly surpassed that of PVA fibers. Maintaining constant fiber content (*V_f_* = 1.0%), a comparison between single and hybrid fiber-reinforced specimens reveals that the residual bond strength of the S05PA05 specimen increased by 55.2% and 58.9% compared to the S0PA10 and S10PA0 specimens, respectively. This signifies a positive synergistic effect between fibers on peak bond strength. Notably, this phenomenon persisted even when the total fiber content was 1.5%. This persistence was attributed to a “pin action” generated by the PVA fiber at the end of the steel fiber, effectively transmitting stress at the end of the steel fiber and enhancing the control capacity of the steel fiber over larger-scale crack development.

#### 3.3.3. Peak Slip Drift

**Figure 8:**
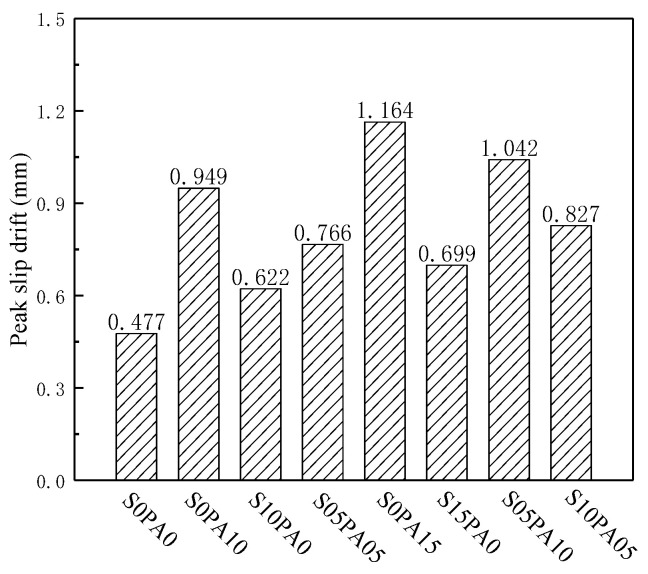
Peak slip drift of specimens.

The incorporation of PVA fibers effectively mitigates stress concentration at the interface of reinforcement and matrix, thereby suppressing crack formation at the junction. During relative sliding between the steel bar and the matrix, the fine-scale PVA fibers bridge across the micro-crack on both sides, impeding micro-crack development. This action results in an augmented mechanical interlock between the steel bar and the matrix, consequently leading to a notable increase in the peak slip displacement of the test block. As depicted in Figure 8, with consistent fiber content, the peak slip displacement of the singly doped steel fiber test block was markedly lower than that of the singly doped PVA fiber test block and the HyFRCC test block. This discrepancy arose from the PVA fiber’s control over micro-cracks due to the larger scale of the steel fiber. Meanwhile, the cracks in the matrix predominantly manifested as micro-cracks before reaching the peak load.

### 3.4. Bond Toughness

**Figure 9:**
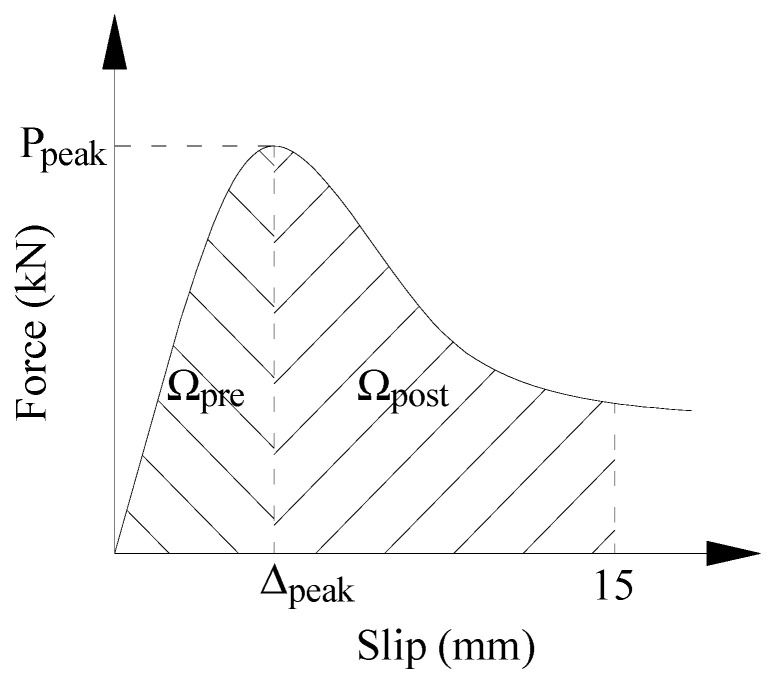
The calculation of equivalent bond toughness.

The bond force–slip curve exhibits two clearly defined regions, separated by the peak loading: pre-peak and post-peak, as illustrated in Figure 9. The region up to the peak loading, as enclosed by the curve, was calculated and termed “pre-peak energy”, Ω_pre_. Additionally, the area encompassed by the curve from peak loading to the 15 mm slip was calculated and designated as “post-peak energy”, Ω_post_. The equivalent bond toughness was calculated as follows:

(2) $$TI_{b}=\frac{\Omega }{A}$$

where *TI*_b_ denotes the equivalent bond toughness index, Ω represents the area enclosed by the force–slip curve at a slip of ∆_s_, and *A* stands for the total contact surface area between the steel rebar and the matrix.

The calculated pre-peak and post-peak equivalent bond toughness, derived from the test results, is presented in Table 6. Both *TI*_b-pre_ and *TI*_b-post_ demonstrated a significant increase with the inclusion of fibers. The improvement in *TI*_b-pre_ resulting from the addition of PVA fibers is obviously better than steel fibers. For *TI*_b-post_, the improvement from the steel fibers was obviously better than from the PVA fibers. Compared with mono-fibrous cementitious composite, the *TI*_b-pre_ and *TI*_b-post_ of HyFRCCs can be effectively improved by the hybridization of fibers with different geometric dimensions when the total fiber content is constant.

### 3.5. Synergy

Constructing a synergy index is essential to gauge the impact of fiber synergy on the performance of HyFRCCs. In this regard, the synergy index proposed by Wang [26] was used to quantitatively evaluate the interplay within the hybrid system. The synergy index was calculated as follows:

(3) $$\left\{\begin{array}{l} R=\frac{S-\left(S_{1}\varphi_{1}+S_{2}\varphi_{2}+S_{3}\varphi_{3}+\cdots \right)}{S_{1}\varphi_{1}+S_{2}\varphi_{2}+S_{3}\varphi_{3}+\cdots }\\ \varphi_{1}+\varphi_{2}+\varphi_{3}+\cdots =1\\ \varphi_{i}=\frac{V_{i}}{V},i=1,2,3,\cdots \end{array}\right.$$

where *R* represents the synergy index, *S* is the mechanical performance parameter of the investigated HyFRCC, *S_i_* (*i* = 1, 2, 3,…) is the corresponding mechanical performance parameter of the mono-fibrous cementitious composite with the *i*th fiber only, *V_i_* is the content of the *i*th fiber in the HyFRCC, *V* is the total content of fibers in the HyFRCC, and $\varphi_{i}$ is the percentage of the *i*th fiber content in the total fiber content.

If *R* = 0, there is no effect of fiber synergy on the HyFRCC. If *R* > 0, there is a positive effect of fiber synergy on the HyFRCC. If *R <* 0, it means that there is a negative effect of fiber synergy.

**Figure 10:**
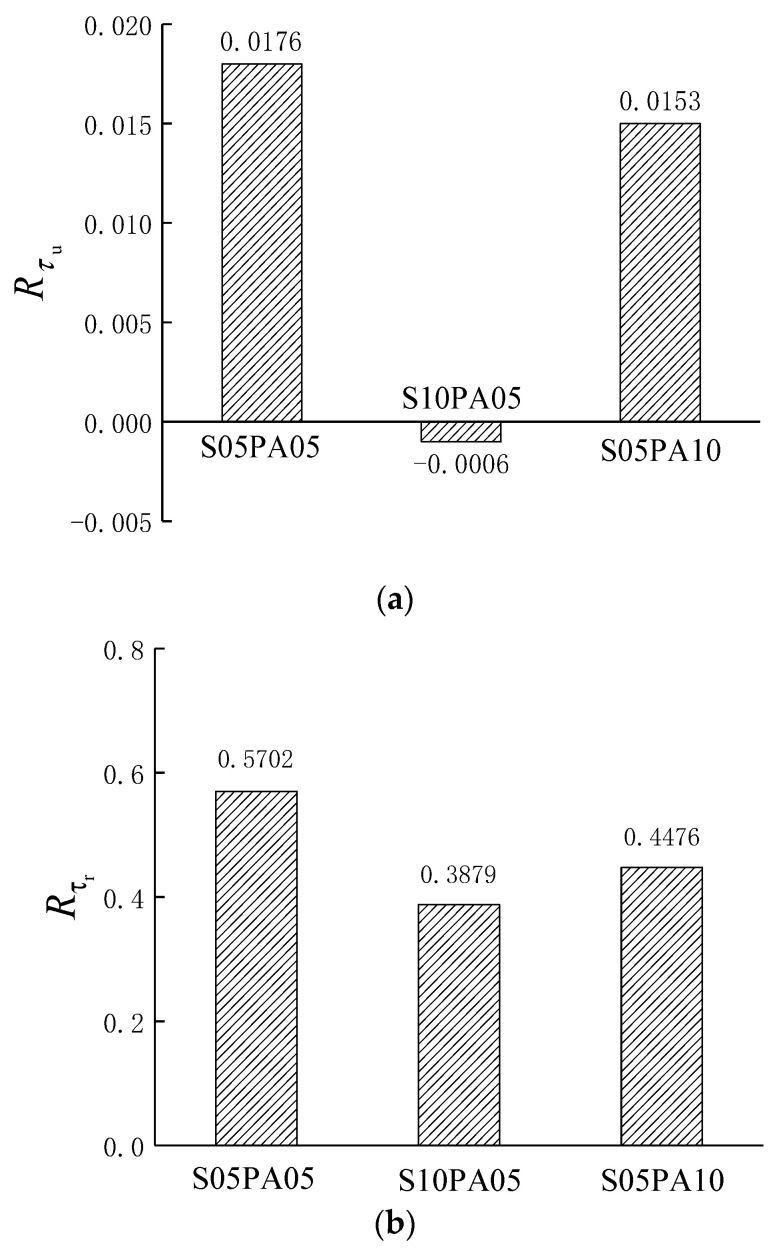
The synergy index of specimens: (**a**) ultimate bond strength, (**b**) residual bond strength.

The synergy indices for comparing bond strength and residual bond strength between the HyFRCCs and steel rebar were calculated and are shown in Figure 10.

A positive synergy effect on bond strength was observed when 0.5% and 1.0% PVA fibers were combined with 0.5% steel fibers, resulting in synergy indices of 0.0176 and 0.0153. However, the synergy effect was not prominent, likely due to the effective restraint of micro-cracks by the PVA fibers. Significantly, the synergy in bond strength diminished with escalating *V*_S_ and *V*_PVA_. The introduction of fibers during mixing introduces more matrix defects, leading to a reduced synergy effect on bond strength. As the number of defects increased with higher *V*_S_, it resulted in a negative synergy effect for the bond strength of the S10PA05 specimen. Before the peak load, the cracks in the test block were mainly micro-cracks of a small scale, and the crack control effect of PVA fiber was due to the steel fiber. Therefore, under the same fiber content (*V_f_* =1.5%), different fiber combinations showed different confounding effects. In terms of residual bond strength, the fiber hybrid effect remained positive. With a constant *V*_S_ of 0.5%, the hybrid effect coefficient decreased from 0.5702 to 0.3879 as the *V*_PVA_ increased from 0.5% to 1.0%. Similarly, when maintaining a constant *V*_PVA_ of 0.5%, the hybrid effect coefficient decreased from 0.5702 to 0.4476 as the *V*_S_ increased from 0.5% to 1.0%. Overall, as the fiber content in the hybrid system increased, the synergistic effect between fibers decreased.

**Figure 11:**
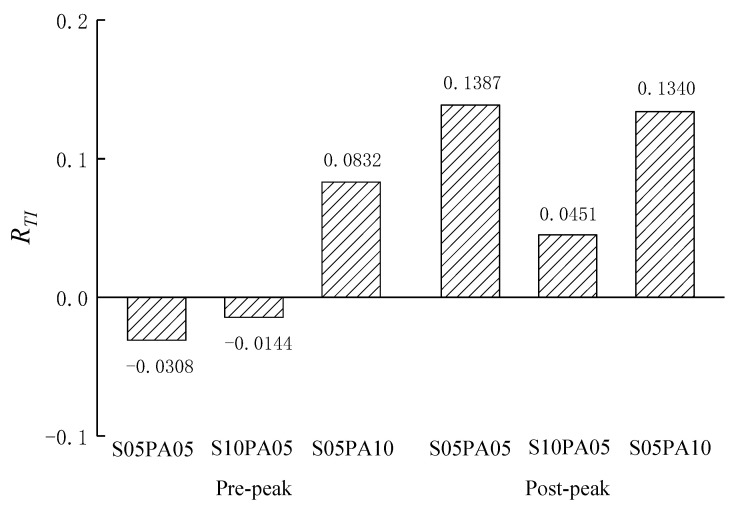
The synergy index for bond toughness.

The synergy indices for pre-peak and post-peak bond toughness were calculated and are illustrated in Figure 11. At a steel fiber content of 0.5%, as the PVA fiber content increased from 0.5% to 1.0%, the synergy index for pre-peak bond toughness transitioned from −0.0308 to 0.0832. Furthermore, with a total fiber content of 1.5%, the synergy index for pre-peak bond toughness evolved from −0.0144 to 0.0832, corresponding to the replacement of a portion of steel fiber with PVA fiber. A negative effect was observed when 0.5% and 1.0% steel fibers were combined with 0.5% PVA fiber. Specifically, with a constant *V*_PVA_ of 0.5%, the synergy index declined as the *V*_S_ increased from 0.5% to 1.0%. The negative synergy effect for pre-peak bond toughness became positive with an increase in PVA fiber content. This is likely due to the low restraint of steel fibers on micro-crack development during the pre-peak stage, introducing defects in the process. Conversely, owing to the effective inhibition of micro-cracks by PVA fibers, a notably positive synergy effect was observed when 0.5% steel fibers were combined with 1.0% PVA fiber. Figure 11 demonstrates positive synergy indices for post-peak bond toughness across all HyFRCC specimens, indicating that the hybridization of steel and PVA fibers was more effective during the post-peak stage. This effectiveness is primarily due to the increasing control exerted by steel fibers on large-scale cracks as the crack size grows, complemented by the pinning effect of PVA fibers on the ends of steel fibers.

## 4. Conclusions

The effect of fiber synergy on the bond behavior of hybrid fiber-reinforced cementitious composite and steel rebar was investigated through the direct pull-out test on eight different specimen groups, each featuring varied combinations and contents of steel and PVA fibers. Based on the direct pull-out test results and the quantitative analysis of fiber synergy, the following main observations were found.

In the case of mono-fibrous cementitious composite mixtures, PVA fiber exhibited greater effectiveness in enhancing bond strength and pre-peak bond toughness compared to steel fiber. Under the same fiber content (*V_f_* = 1.5%), specimens reinforced with PVA fiber exhibited a 4.15% increase in peak bond strength and a 66.77% increase in pre-peak bond toughness relative to specimens solely reinforced with steel fiber. Nevertheless, steel fiber surpassed PVA fiber in post-peak bond toughness. At an identical fiber content (*V_f_* = 1.5%), the post-peak bond toughness of specimens reinforced with steel fiber outperformed that of PVA fiber-reinforced specimens by 3.27%.

For PVA–steel HyFRCCs, both positive and negative synergy effects were observed, yet the overall synergy effect was not pronounced. Notably, when the total fiber content was low, a positive synergy effect on bond strength was evident. However, as the fiber content increased, the synergy effect diminished.

The synergy effect on bond toughness in PVA–steel HyFRCCs was not consistently positive for all investigated fiber combinations. At a steel fiber content of 0.5%, as the PVA fiber content increased from 0.5% to 1.0%, the confounding effect coefficient associated with bonding toughness before the peak shifted from −0.0308 to 0.0832. Furthermore, when the total fiber content reached 1.5%, the confounding effect coefficient linked to pre-peak bond toughness underwent a transformation from −0.0144 to 0.0832 due to the substitution of a portion of steel fiber with PVA fiber. The negative synergy effect for pre-peak bond toughness became positive with an increase in PVA fiber content. Conversely, the synergy effect for post-peak bond toughness was positive for all investigated fiber combinations.

While this study has unveiled positive and negative synergies between hybrid fibers and HyFRCCs in terms of bond–slip properties, it is imperative to explore analogous synergies between HyFRCCs and corrosion resistance, along with other durability characteristics. This exploration is essential for a comprehensive understanding of how hybrid fibers can enhance both the mechanical properties and durability of HyFRCCs. Subsequently, the optimization of the HyFRCC design should be further pursued.

## Figures and Tables

**Table 1:** Mixture design (kg/m^3^).

Cement	Fly Ash	Sand	Water	w/b
530	753	467	404.5	0.315

Note: w/b: water-to-binder ratio.

**Table 2:** The properties and geometry of the steel fibers.

Length (mm)	Diameter (mm)	Tensile Strength (MPa)	Density (g/cm^3^)	Type
36	0.6	≥600	7.8	Crimped steel

**Table 3:** The properties and geometry of the PVA fibers.

Length (mm)	Diameter (μm)	Tensile Strength (MPa)	Elongation (%)	Modulus (GPa)	Density (g/cm^3^)
12	40	1600	6	40	1.3

**Table 4:** Volume fraction of fibers in different specimen groups.

Fiber Volume (%)	Specimen Group							
	S0PA0	S0PA10	S10PA0	S0PA15	S15PA0	S05PA05	S05PA10	S10PA05
*V*_S_	0	0	1.0	0	1.5	0.5	0.5	1.0
*V*_PVA_	0	1.0	0	1.5	0	0.5	1.0	0.5

Note: *V*_S_: steel fiber content, *V*_PVA_: PVA fiber content. Nomenclature: S05PA10—“S” denotes the steel fiber nomenclature, and “PA” denotes the PVA fiber nomenclature. The numerical values 05 and 10 correspond to the volume fractions of 0.5% and 1.0% for steel fiber and PVA fiber, respectively, within the cement mortar.

**Table 5:** Test results of specimens.

Specimen Group	S0PA0	S0PA10	S10PA0	S0PA15	S15PA0	S05PA05	S05PA10	S10PA05
$\tau_{u}$ (MPa)	11.71	15.33	14.82	16.56	15.90	15.34	16.59	16.11
$s_{u}$ (mm)	0.477	0.949	0.622	1.164	0.699	0.766	1.042	0.827
$\tau_{r}$ (MPa)	1.04	2.99	2.92	3.58	3.43	4.64	5.11	4.83

Note: $\tau_{u}$: ultimate bond strength. $s_{u}$: peak slip drift (the slip displacement corresponding to the ultimate bond strength). $\tau_{r}$: residual bond strength (the bond strength corresponding to a slip drift of 15 mm).

**Table 6:** Volume fraction of fibers in different specimen groups (%).

Specimen Group	Ω_pre_	Ω_post_	*TI*_b-pre_	*TI*_b-post_
	(kN·mm)		(N·mm/mm^2^)	
S0PA0	15.03	196.64	4.78	62.59
S0PA10	41.17	304.46	13.10	96.91
S10PA0	26.13	304.03	8.32	96.78
S0PA15	52.55	348.76	16.73	111.01
S15PA0	31.58	360.14	10.05	114.64
S05PA05	32.59	346.46	10.38	110.28
S10PA05	38.01	372.40	12.10	118.54
S05PA10	49.34	399.80	15.71	127.26

## Data Availability

The data presented in this study are available on request from the corresponding author.
